# Critical values for dimensional parameters of mesotheliomagenic mineral fibers: evidence from the dimensions and rigidity of MWCNT

**DOI:** 10.3389/ftox.2025.1568513

**Published:** 2025-04-22

**Authors:** Ann G. Wylie, Andrey A. Korchevskiy

**Affiliations:** ^1^ Department of Geology, University of Maryland, College Park, MD, United States; ^2^ Chemistry & Industrial Hygiene, Inc., Lakewood, CO, United States

**Keywords:** mesothelioma, carbon nanotubes, rigidity, EMP, EMPA, asbestos, cleavage fragments

## Abstract

MWCNT (multi-walled carbon nanotubes) used in 72 animal instillation or inhalation studies were classified by average length, average width, Young’s modulus, Rigidity Index (RI), and potency for mesothelioma in animals. The RI is based on the Euler buckling theory. MWCNT that induce mesothelioma have average lengths >2 µm and widths >37 nm, and average RI > 0.05 (µm^2^ x GPa x 10^4^). Many noncarcinogenic MWCNT materials have RI < 0.05 and lack biological rigidity. In comparison, Elongate Mineral Particle (EMP) populations with one exception have RI > 0.05. Mineral particles likely to have RI < 0.05 include chrysotile fibrils with lengths >5 μm, amosite and crocidolite fibers with widths <60 nm, and sheet silicate fibers with widths <200 nm. The product of percent EMPA, average RI, and biosolubility among silicates correlates with known mesothelioma potency. The derived models reproduce published values of R_M_ with high statistical significance (P < 0.05). Average RI, length, and width are critical parameters for mesotheliomagenicity for both MWCNT and EMPA mineral fiber.

## 1 Introduction

Not all types of elongate mineral and mineral-like^1^ particles elevate risk of mesothelioma in exposed populations. Stanton et al. (1981), Pott et al. (1987), Berman and Crump (2003), Berman and Crump (2008b), Lippmann (2014), Gamble and Gibbs (2008), Wylie et al. (2020), Korchevskiy and Wylie (2021), Korchevskiy and Wylie (2022), Korchevskiy and Wylie (2023), and Wylie and Korchevskiy (2023) have concluded that durable elongate particles that are capable of inducing mesothelioma express specific frequency distributions of lengths and widths that distinguish them from equidimensional particles and other elongate mineral particles (EMPs) consistent with the fiber pathogenicity paradigm (FPP) (Nel, 2023; Murphy et al., 2021a). According to FPP, mesothelioma can be caused by any durable elongate material whether mineral or man-made, provided the material conforms to specific dimensional characteristics. Determining critical values for dimensional parameters that separate carcinogenic vs non-carcinogenic EMPs is essential for protecting health because it enables exposure assessments to be designed to maximize detection of carcinogenic fiber. This is particularly important for certain environmental and occupational potential exposures to rock dust.

Determining the critical dimensional parameters for an aerosol, where exposure monitoring can take place, is complicated. First, not all particles in an aerosol can be inhaled. Second, the dimensional characteristics of particles in an aerosol vary within and between exposures. Third, only a subset of the inhaled aerosol passes through the alveolar openings to access the lung parenchyma and only a subset of these will translocate outside the lung to the pleura.

Rigidity, an outgrowth of dimension and structural strength, is a characteristic that reflects the ability of an object to withstand mechanical forces without bending. Biologically rigid particles would not lose their integrity while in mechanical contact with tissue and cell structures (Manning, 2024). Rigidity may play an important role in carcinogenicity because it affects the clearance of fiber.

Carbon nanotubes (CNTs) research provides an opportunity to examine both rigidity and dimensions and their role in inducing mesothelioma from a large number of animal inhalation and implantation studies. The multiwalled carbon nanotubes used had a wide range of both width, length, and strength. From these studies, it is now generally accepted that a) dimensions affect rigidity, b) biological rigidity is necessary for carbon nanotubes to induce a carcinogenic response, and c) it is the loss of rigidity by fiber narrower than about 40 nm that results in its loss of carcinogenic potency. In recognition of these studies, in 2021 in the EU, a harmonized classification and labelling system for Multi-Walled Carbon Nanotubes (MWCNT) which considers any tube with diameter ≥30 nm,<3 um, and longer than 5 µm to be a carcinogen (BAuA, 2021) was proposed under the CLH regulation. In addition, the FPP has been expanded to include rigidity. While nonrigid materials may not be carcinogenic, this does not mean that they are nontoxic. Rather, they should be evaluated separately (Murphy et al., 2021b). For example, narrow “tangled” MWCNT may form aligned bundles, whose potential for mesotheliomagenicity is unknown.

The commonality of animal response to certain multiwalled carbon nanotubes and asbestos makes a more detailed comparison of dimensions of these two groups of materials worthwhile to advance understanding about the critical dimensions of mineral fiber.

## 2 Purpose

In this paper we will review the literature describing animal studies with MWCNTs to better understand the relationship between dimension, rigidity, and mesotheliomagenicity among this set of materials and the experimental outcomes. We will also compare naturally occurring elongate silicate particle populations from the dimensional database containing currently 567,000 records [see Wylie et al. (2022) regarding the database organization and sources of data] to carbon nanotubes by average dimension and rigidity. Because of the lattice strength and size of most silicate EMPs, silicates have generally been considered rigid, with the exception, perhaps, of chrysotile. However, there is a great range in the physical properties, sizes, and structures among silicate EMPs. Given the importance of rigidity in explaining the mesotheliomagenicity of MWCNT, a quantitative method for calculating rigidity and relating that quantity to mesotheliomagenicity in general is needed. Such a measure would generally confirm the biological rigidity of most silicate EMPs; it would also enable identification of biologically weak mineral fiber; and perhaps it could be used to assess newly discovered HARNs, limiting the need for extensive *in vivo* studies and assisting regulatory agencies in assessing risk of all types of fiber. Furthermore, given a quantitative nature, rigidity can be incorporated into QSAR modelling that relates physical and chemical properties of EMPs to carcinogenicity.

In this paper, we will compare the quantitative relationships between centrality measures of **rigidity, dimension, lattice strength**, and **carcinogenicity** of elongated mineral particle (EMP) populations from our extensive database to populations of carbon nanotubes that have been used in animal inhalation and implantation experiments and have caused responses that range from no affect to inflammation, mesothelioma, lung cancer, and lung fibrosis.^2^ We will attempt to demonstrate how these relationships reflect the mesotheliomagenic potency of EMPs.

## 3 Materials and methods

### 3.1 Properties of carbon nanotubes

For more than 20 years, multi-walled carbon nanotubes (MWCNT) have been manufactured and used in a variety of industrial applications. Because of their similarities to asbestos in physical properties, their potential to cause mesothelioma has been evaluated in both animal and cell studies.

Dimensions of MWCNT are normally well-controlled by the manufacturing process such that the ranges in length and width in specific products are usually small (in comparison to elongated silicate mineral populations). However, there is a great variability among manufacturing processes, which result in a wide range of both average length and average width among available materials. Average widths range from 1 to 200 nm and average lengths range from nanometers to micrometers. Within the group are both straight and tangled nanotubes. The physical properties and their potential toxicity vary significantly across this range of widths.

Some MWCNT are manufactured with an arc-discharge process, which can produce fibers with a highly crystalline structure and few defects. Another method, chemical vapor deposition (CVD), produces tubes with a higher number of defects and lowered strength, but there are other methods as well. The internal lattice strength of carbon nanotubes, as measured by Young’s modulus, generally decreases as chemical impurities and associated defects increase, effecting surface properties and bioreactivity. Separating out these effects can be difficult.

There is also a range in composition, and chemical impurities can be as high as 30% amorphous carbon, metals, alumina, and silica, with only very difficult fabrication techniques producing carbon nanotubes with a purity approaching 99% (Fubini et al., 2011). No association between impurities and carcinogenicity of carbon nanotubes has been found (Murphy et al., 2013) although inflammation may be influenced by composition. In their recent review of the factors that influence the carcinogenicity of high aspect ratio nanoparticles (HARNS), Murphy et al. (2021b) do not include composition as a factor.

Unlike silicate fibers, carbon nanotubes are conductors of heat and electricity along their lengths. Also, unlike most silicate fibers which carry a negative charge, the surfaces of pristine MWCNT carry a strong positive charge. The widths of single carbon nanotubes can be of similar size to narrow silicate fibrils, although many are much smaller. Silicate fibers are denser than carbon fibers and they are generally hydrophilic while carbon nanotubes are hydrophobic. It is well established that phagocytosis and endocytosis are dependent on size, shape, net charge, stiffness, hydrophobic vs hydrophilic, and texture so there may be differences in the biological response to MWCNT and silicate fibers based on factors other than dimension (Frank et al., 2016; Nagai and Toyokuni, 2012; Funahashi et al., 2015; Dymacek et al., 2018; Augustine et al., 2020; Donaldson et al., 2013; Dumit et al., 2024). In this paper, however, we only examine the relationships among lattice strength as measured by Young’s modulus, length and width.

Though differences between carbon nanotubes and elongate mineral particles are obvious and pronounced, there is a potential significant overlap in the apparent ability of both to produce mesothelioma. In 2014, the International Agency for Research on Cancer (IARC) classified the long, rigid, needle-shaped Mitsui-7, as possibly carcinogenic to humans (Group 2B). In 2022, Barbarino and Giordano (2021) noted that CNTs induce a sustained inflammatory response, oxidative stress, fibrosis, and histological alterations. The development of mesothelial hyperplasia, mesothelioma, and lung tumors have also been described *in vivo*. The data support a strong inflammatory potential of CNTs, similar to that of asbestos, and provide evidence that CNT exposure led to molecular alterations known to have a key role in mesothelioma onset.

Because some are known carcinogens but others are not (Grosse et al., 2014), carbon nanotube studies provide an opportunity to test some hypotheses about the properties of carcinogenic fibers generally, despite the differences between carbon nanotubes and silicate fibers. It is likely that physical properties, not surface chemistry, are of first order importance in predicting carbon nanotube carcinogenicity so they provide a special opportunity to study a material that is relatively homogeneous chemically, but exists in samples of different average width, length, and strength of the atomic structure.

### 3.2 Establishing rigidity

Because of the work on carbon nanotubes, the set of factors affecting the carcinogenic potential of fiber has been expanded to include **rigidity**, incorporating what has been learned from animal research with carbon nanotubes into the overarching paradigm for mesotheliomagenic fibers (Lee et al., 2018; Nagai et al., 2011; Murphy et al., 2021b; Fortini et al., 2020; Zhu et al., 2016). Only rigid nanotubes have been implicated in animal carcinogenicity.

Rigid fibers are more difficult for macrophages to engulf (Fortini et al., 2020), and they have an enhanced capacity to migrate with fluids as their long axis remains aligned with the direction of flow so the effective aerodynamic diameter will closely align with actual fiber width, unlike fibers that are highly flexible or agglomerate readily. The higher the rigidity, the greater the crystallinity, surface charge, and conductivity along the tube length. Rigidity of CNTs shows a strong positive correlation with inflammogenicity potential (Lee et al., 2018) as well as carcinogenicity [reviewed by Nel (2023)]. MWCNT-7 is classified by IARC as a Group 2B carcinogen (Grosse et al., 2014). It is distinguished by the rigidity of its fibers, with a Young’s modulus exceeding 5,000 GPa (Zhu et al., 2016).

The narrowest carbon nanotubes lack rigidity and are often referred to as “tangled” when observed under the microscope; average lengths are sometimes not reported because of the difficulty in measuring curved intertwined fibers. Fibers with widths below 15 nm are not known to cause mesothelioma and fibers with widths greater than 200 nm have not been manufactured (BAuA, 2021). As mentioned earlier, BAuA (EU CLH Proposal) proposed a 30 nm minimum for a carcinogenic fiber (BAuA, 2021).

From the human experience, the most potent known amphibole asbestos carcinogen (per fiber-year exposure) is crocidolite from Cape South Africa (SA) and Australia. The fiber from these two locations is remarkably similar and crocidolite from both locations is usually thought of as a “rigid” fiber. The most widely used asbestos, chrysotile, is described as soft and lacking rigidity in hand specimens, with individual fibrils that can be many millimeters in length. Long fibers can be easily woven. Despite the differences in macroscopic rigidity between chrysotile and crocidolite (and other silicate fibers), the rigidity of mineral fiber has not been quantitatively determined outside a few calculations for asbestos.

Biological rigidity might be defined as the stiffness necessary to pierce the cell membrane when subject to biological forces, such as from the membrane of a macrophage attempting to engulf a fiber contributing to frustrated phagocytosis. One mechanism that could cause the DNA damage that eventually leads to cancer in epithelial or mesothelial cells is physical interaction with DNA by a fiber within the nucleus (Nagai and Toyokuni, 2012; Fatkhutdinova et al., 2024; Møller and Jacobsen, 2017) and rigidity may be important in enhancing membrane piercing. It has also been shown that rigidity positively correlates with inflammogenic potential (Lee et al., 2018). If rigidity is a necessary characteristic of mesotheliomagenic fiber, tangled MWCNT fiber would not qualify. As discussed below, rigidity can be defined mathematically as a function of width, length, and crystal structural strength as manifest in Young’s modulus parallel to the fiber axis. The widths of multi-walled carbon nanotubes, chrysotile, and crocidolite that can be considered the threshold width for biological flexural rigidity have been estimated as 37–44, 60, and 56 nm respectively by Broβell et al. (2020).

### 3.3 Animal studies: materials used and outcomes

Reports of the outcomes of 72 animal inhalation and implantation experiments with MWCNT samples reported in 55 publications were compiled along with the average length and width for each MWCNT used, as reported by the investigator or as determined from published dimensional parameters, assuming normal distributions. The studies selected included all of those reviewed by Kuempel et al. (2017), Witkowska et al. (2022), as and Kobayashi et al. (2017) well as those identified on a PubMed search for key words animal studies and carbon nanotubes, and each of the reported responses: mesothelioma, lung cancer, fibrosis, and inflammation for which lung tissue was evaluated and dimensional data on MWCNT were available. Each material was assigned to one of four categories as reported by the investigators as a statistically significant outcome following exposure: 1) mesothelioma, including those also reporting lung cancer or fibrosis, 2) lung cancer, including those also reporting fibrosis, 3) fibrosis, and 4) those reporting none of the above. Inflammation and other tissue effects were commonly reported in most studies but are not considered separately in this analysis.

The data and sources are reported in Table 1. Further studies may expand the list of experiments and refine the boundaries we suggest in this paper, but the internal logic of the data we analyzed makes us confident that changes to our general conclusions are unlikely.

**TABLE 1:** Average dimensions, Young’s modulus and Rigidity Indices of MWCNT by outcome.

MWCNT	Way of exposure/application	Central tendency (average length) (µm)	Central tendency (average width) (nm)	Young’s modulus (GPa)	Rigidity index (µm^2^ x GPa x 10^4^)	Source
a. Mesothelioma (lung cancer may be reported)						
NT 50a	Intraperitoneal	5.3	50	5,120	5.511	Nagai et al. (2011)
NT 145	Intraperitoneal	4.6	143	1,200	114.7	Nagai et al. (2011)
Mitsui-7	Peritoneal injection	2.8	89	5,120	198.2	Takagi et al. (2008)
Mitsui-7	Intrascrotal injection	2.8	89	5,120	198.2	Sakamoto et al. (2009)
Mitsui-7	Intratracheal instillation	4.8	87.5	5,120	64.5	Numano et al. (2019)
CNT-7 Mitsui-7	Intraperitoneal	7.1	75	5,120	15.5	Huaux et al. (2016)
CNT-7 short (Mitsui-7)	Intraperitoneal instillation	2.8	75	5,120	100	Huaux et al. (2016)
Long MWCNT	Pleural instillation	25.5	165	1,200	6.46	Chernova et al. (2017)
MWCNT A	Intraperitoneal	8.6	85	1,200	4.1	Reamon-Buettner et al. (2024), Rittinghausen et al. (2014)
MWCNT B	Intraperitoneal	9.3	62	1,200	0.992	Reamon-Buettner et al. (2024), Rittinghausen et al. (2014)
MWCNT C	Intraperitoneal	10.2	40	1,200	0.143	Reamon-Buettner et al. (2024), Rittinghausen et al. (2014)
MWCNT D	Intraperitoneal	7.9	37	1,200	0.174	Reamon-Buettner et al. (2024), Rittinghausen et al. (2014)
MWCNT-7	Intratracheal	5.1	85	5,120	49.71	Hojo et al. (2022)
M-CNT	Intraperitoneal	6.65	67	1,200	2.645	Sakamoto et al. (2018)
N-CNT	Intraperitoneal	5.5	59.2	1,200	2.36	Sakamoto et al. (2018)
WL-CNT	Intraperitoneal	7.3	71	1,200	2.77	Sakamoto et al. (2018)
SDI-CNT	Intraperitoneal	4.5	177	1,200	281.36	Sakamoto et al. (2018)
MWCNT-N unfiltered	Transtracheal interpulmonary spray	4.2	55	1,200	3.01	Suzui et al. (2016)
MWCNT-N filtered	Transtracheal interpulmonary spray (TIPS)	2.6	55	1,200	7.86	Suzui et al. (2016)
MWCNT 7	Intratrachael instillation	8.79	76.49	5,120	10.98	Saleh et al. (2022)
Mitsui-7	Inhalation	3.5	56	5,120	19.88	Sargent et al. (2014), McKinney et al. (2009)
b. Lung Cancer (fibrosis may be reported)						
NRCWE006 (Mitsui-7)	Inhalation	5.5	87	5,120	46.91	Kasai et al. (2016)
MWCNT-B	Intratracheal instillation	1.04	7.4	1,200	0.016	Saleh et al. (2020)
MWCNT	Intratracheal instillation	7.71	13.5	1,200	0.003	Yu et al. (2013)
c. No cancer reported (lung fibrosis recorded)						
NM 403 CCV	Intratracheal instillation	0.4	12	1,200	0.752	Bubols et al. (2023)
MWCNT	Inhalation	25.25	30	1,200	0.007	Ryman-Rasmussen et al. (2009)
MWCNT	Pharyngeal Aspiration	3.86	49	5,120	9.583	Porter et al. (2010)
MWCNT	Intratracheal instillation	5	88	1,200	13.925	Aiso et al. (2010)
MWCNT-L	TIPS	8	150	1,200	45.92	Xu et al. (2014)
VGCFtm-H(CVD)	Intratracheal instillation	9	75	160	0.302	Numano et al. (2021)
Mitsui-7	Intratracheal instillation	5.7	74	5,120	22.859	Rahman et al. (2017)
NM 401	Intratracheal instillation	4	67	1,200	7.31	Rahman et al. (2017), Bubols et al. (2023), Gaté et al. (2019)
MWCNT-L	Intratracheal instillation	10	15	1,200	0.003	Chen et al. (2014)
MWCNT	Inhalation	0.33	11	160	0.104	Ellinger-Ziegelbauer and Pauluhn (2009), Pauluhn (2010)
MWCNT	Pharnygeal aspiration	3.86	49	1,200	2.246	Mercer et al. (2011)
MWCNT	Inhalation	0.33	11	160	0.104	Pauluhn (2010)
MWCNT	Intratracheal instillation	24	20	1,200	0.002	Cesta et al. (2010)
d. No cancer or fibrosis reported						
DWCNT	Pharyngeal instillation	0.52	22	1,200	5.029	Crouzier et al. (2010)
MWCNT-WS	Intraperitoneal	1.25	44.5	1,200	14.57	Sakamoto et al. (2018)
MWCNT-SD2	Intraperitoneal	3	13.5	1,200	0.021	Sakamoto et al. (2018)
MWCNT-T	Intraperitoneal	0.732	35.8	1,200	17.795	Sakamoto et al. (2018)
MWCNT+	Intraperitoneal	0.7	11.3	1,200	0.193	Muller et al. (2009)
MWCNT-	Intraperitoneal	0.7	11.3	1,200	0.193	Muller et al. (2009)
MWCNT	Intraperitoneal	1.5	20	1,200	0.413	Varga and Szendi (2010)
NTtng1	Intraperitoneal	3	15	1,200	0.033	Nagai et al. (2011), Nagai et al. (2013)
NM 400	Pulmonary instillation	0.85	11	160	0.016	Knudsen et al. (2019)
NM 401	Pulmonary instillation	4	67	160	0.0975	Knudsen et al. (2019)
NM 402	Pulmonary instillation	1.4	11	160	0.006	Knudsen et al. (2019)
NM 403	Pulmonary instillation	0.4	12	160	0.1	Knudsen et al. (2019)
Nanotechcenter	Aspiration	8.5	11.5	1,200	0.002	Khaliullin et al. (2015)
NM403	Inhalation and intratracheal instillation	0.4	12	160	0.1	Gaté et al. (2019)
MWCNT-A	Intratracheal instillation spray	6.39	150	1,200	71.97	Saleh et al. (2020)
SNT	Pharyngeal aspiration	1.5	25.7	1,200	1.125	Murphy et al. (2013)
TNT	Pharyngeal aspiration	3	14.84	1,200	0.031	Murphy et al. (2013)
MWCNT(CVD)	Inhalation	10	15	160	0.00039	Mitchell et al. (2007)
NC7000	Intratracheal instillation	0.4	8.3	1,200	0.17218	Fujita et al. (2022)
NM401	Inhalation	4	67	160	0.9748	Seidel et al. (2021)
NM403	Inhalation	0.4	12	160	0.1003	Seidel et al. (2021)
MWCNT(JEIO)	Inhalation	1.5	7.5	1,200	0.0082	Kim et al. (2020)
Mitsui-7	Pharyngeal aspiration	4.46	58.5	5,120	3.418	Lim et al. (2020)
MWCNT	Inhalation	20	15	1,200	0.000735	National Toxicology Program (2019)
NC 7000	Inhalation	5.5	10	1,200	0.00192	Ma-Hock et al. (2009)
MWCNT(Baytube)	Intratracheal instillation	1.3	35	1,200	5.1547	Elgrabli et al. (2008)
CVD solution	Inhalation and instillation	0.94	44	160	3.283	Morimoto et al. (2012)
CVD aerosol	Inhalation and instillation	1.1	3	160	0.00005	Morimoto et al. (2012)
CVD	Intratracheal instillation	7	90.5	160	1.059564	Park et al. (2009)
MWCNT-7(sonicated)	Instillation	1.5	60	5,120	142.66	Kobayashi et al. (2010)
MWCNT-S	TIPS	3	15	1,200	0.03265	Xu et al. (2014)
T/CNT	Aspiration	12.5	31	1,200	0.03431	Rydman et al. (2015)
MWCNT-S	Intratracheal instillation	0.5	15	1,200	1.1755	Chen et al. (2014)
MWCNT (NM 402)	Inhalation	1.1	7.5	160	0.002	Pothmann et al. (2015)
MWCNT	Inhalation	20	25	1,200	0.00567	Silva et al. (2014)
MWCNT	Intratracheal aspiration	20	31	1,200	0.0134	Han et al. (2010)

Table 1 also shows the Young’s modulus we assigned to each experimental material. The Young’s modulus for Mitsui-7 was measured by Zhu et al. (2016) as 5,120 GPa. This value was used if the materials were comparted as similar to Mitsui-7. The Young’s modulus for catalytic chemical vapor deposition (CVD) MWCNT was measured by Fortini et al. (2020) using dynamic scanning electron microscopy. They report values between 30 and 160 GPa. For purposes of this study, it was assumed to be 160 GPa. If no indication as to the manufacturing process of the MWCNT was provided in the study, following the work of Fortini et al. (2020) who report arc discharge formed CNT as 200–1,200 GPa, we assume a value of 1,200 GPa. Because the estimate of Young’s modulus from Table 1 are among the highest for each group, if there is an error in the rigidity estimates that directly depend on them, the error will overestimate rigidity. An error in rigidity of an order of magnitude, for example, would be expected if a material assigned a Young’s modulus of 1,200 actually has a Young’s modulus of 160 GPa. While this may seem like a large error, the range in rigidity for MWCNT and silicate EMPs extends over 10 orders of magnitude.

### 3.4 Elongate fragments and fibers

Dimensional data from the dimensional database for 63 different occurrences of elongate mineral particles (EMP) were used in conjunction with estimates of Young’s modulus parallel to elongation to evaluate the dimensions and rigidity of elongate silicate particles (Tables 2–4). Average length, width, Rigidity Index and their standard deviations for EMPs are provided in three parts. Table 2 is for short EMPs (length ≤5 µm); Table 3 is for long EMP (length >5 µm) with width ≤0.15 µm (EMPA); and Table 4 is for long EMP without the EMPA component.

**TABLE 2:** Average dimensional characteristics of short EMPs (Length/Width ≥ 3) with Length ≤5 µm.

Location	Mineral type	Length (µm)		Width (µm)		Rigidity index (µm^2^ x GPa x 10^4^)	
		Mean	StDev	Mean	StDev	Mean	StDev
Shadwell Quarry, VA	Actinolite	3.061	0.061	0.702	0.011	7,671.89	262.00
Boulder City, NV	Actinolite	3.325	0.328	0.210	0.062	51.04	1,410.93
Fairfax, VA	Actinolite	3.331	0.082	0.712	0.015	1708.29	352.73
San Bernardino, CA	Actinolite	2.974	0.033	0.656	0.006	6,979.34	140.39
Enoree, SC	Actinolite	2.089	0.088	0.259	0.017	677.40	378.78
Rock Hill Quarry, PA	Actinolite	2.274	0.068	0.237	0.013	180.64	293.40
Devon, England	Actinolite	3.209	0.036	0.167	0.007	41.11	154.41
Transvaal, SA	Amosite	2.723	0.011	0.314	0.002	427.74	45.74
India	Anthophyllite	3.247	0.076	0.476	0.014	775.40	329.15
Sweden	Anthophyllite	3.108	0.104	0.707	0.020	7,944.39	446.18
Finland	Anthophyllite	2.714	0.081	0.291	0.015	332.86	347.35
Russia	Anthophyllite	2.977	0.293	0.292	0.055	154.72	1,261.97
Balangero, Italy	Balangeroite	2.575	0.100	0.359	0.019	6,996.75	430.33
Québec, Canada	Chrysotile	1.438	0.005	0.141	0.001	494.95	21.35
Calidria, CA	Chrysotile	1.662	0.040	0.085	0.008	101.46	173.02
Swift Creek, WA	Chrysotile	1.665	0.054	0.154	0.010	645.63	230.79
Balangero, Italy	Chrysotile	2.634	0.159	0.116	0.030	116.12	684.40
Armley, England	Chrysotile	1.657	0.071	0.065	0.013	23.91	304.29
Australia	Crocidolite	1.785	0.011	0.120	0.002	90.45	48.09
Bolivia	Crocidolite	3.599	0.081	0.340	0.015	473.32	350.01
Cape, SA	Crocidolite	1.729	0.011	0.123	0.002	92.19	46.90
Oregon, ND	Erionite	3.115	0.018	0.224	0.003	853.37	78.94
Karain, Turkey	Erionite	1.537	0.024	0.153	0.005	763.55	103.07
Transvaal, SA	Ferro-actinolite	2.241	0.032	0.287	0.006	349.74	136.48
New York	Fibrous talc	2.626	0.096	0.252	0.018	13.02	411.61
Italy	Fluoro-edenite	2.833	0.073	0.431	0.014	3,066.80	315.49
Calaveras Dam, CA	Glaucophane	2.214	0.009	0.313	0.002	539.85	40.82
Homestake, SD	Grunerite	2.878	0.015	0.746	0.003	10653.08	63.83
Portugal	Grunerite	2.423	0.071	0.509	0.013	4,828.08	304.29
Finland	Grunerite	3.092	0.010	0.774	0.002	10310.28	42.53
Taconite mine, MN	Grunerite & Actinolite	2.985	0.108	0.697	0.020	8,658.32	467.08
Goonyella Mine, Australia	Hornblende	3.421	0.185	0.519	0.035	885.92	798.14
Libby, MT	Na-Ca amphibole	2.780	0.020	0.306	0.004	306.30	85.93
Long Valley Creek, CA	Riebeckite	2.936	0.060	0.607	0.011	6,308.09	259.22
Pilbara, Australia	Riebeckite	3.325	0.008	0.580	0.001	4,132.35	34.19
Pikes Peak, CO	Riebeckite	3.283	0.069	0.803	0.013	12349.70	298.28
El Dorado Hills, CA	Tremolite	2.999	0.024	0.556	0.005	1,139.04	103.98
Udaipur, India	Tremolite	2.038	0.074	0.323	0.014	652.32	320.54
Balmat, NY	Tremolite	2.868	0.098	0.767	0.018	10423.68	420.66
Lone Pine, CA	Tremolite	2.797	0.042	0.238	0.008	190.52	182.53
Miners Bay, Canada	Tremolite	2.006	0.096	0.292	0.018	898.44	413.82
Barstow, CA	Tremolite	3.012	0.027	0.270	0.005	601.23	114.44
Gouverneur, NY	Tremolite	2.904	0.097	0.675	0.018	7,988.87	418.34
Madagascar	Tremolite	2.859	0.013	0.685	0.002	6,999.81	56.44
Shinness, Scotland	Tremolite	1.317	0.007	0.297	0.001	1935.41	29.08
Dornie, GB	Tremolite	1.252	0.007	0.305	0.001	2,215.51	31.16
Ala di Stura, Italy	Tremolite	1.337	0.011	0.282	0.002	695.96	47.00
Swansea Lab	Tremolite	1.273	0.011	0.286	0.002	1727.38	48.76
Jamestown, CA	Tremolite	1.190	0.009	0.241	0.002	565.68	37.75
Korea	Tremolite	1.695	0.024	0.212	0.005	283.00	105.38
Canada	Tremolite	3.033	0.057	0.555	0.011	4,668.40	247.49
Metsovo, Greece	Tremolite	2.802	0.083	0.150	0.016	38.58	356.94
Falls Village, CT	Tremolite	2.160	0.068	0.329	0.013	1,310.17	292.61
Eastern New York	Tremolite	2.882	0.088	0.144	0.017	60.28	377.09
Québec, Canada	Tremolite	2.074	0.067	0.336	0.013	1,612.78	288.76
New York	Wollastonite	3.242	0.086	0.721	0.016	8,774.88	372.14
Synthetic	Na-clinojimthompsonite	2.196	0.066	0.131	0.012	88.28	284.33

**TABLE 3:** Average dimensional characteristics of EMPA with L > 5 µm and W ≤ 0.15 µm.

Location	Mineral type	Length (µm)		Width (µm)		Rigidity index (µm^2^ x GPa x 10^4^)	
		Mean	StDev	Mean	StDev	Mean	StDev
Transvaal, SA	Amosite	9.386	1.113	0.113	0.001	1.348	0.078
Transvaal, SA	Ferro-actinolite	5.778	8.492	0.130	0.011	2.350	0.597
Udaipur, India	Tremolite	65.000	18.990	0.100	0.025	0.009	1.335
Lone Pine, CA	Tremolite	8.753	2.193	0.115	0.003	1.243	0.154
Fairfax, VA	Actinolite	6.860	18.990	0.100	0.025	0.730	1.335
India	Anthophyllite	6.433	7.753	0.100	0.010	0.932	0.545
Finland	Anthophyllite	7.054	5.482	0.121	0.007	1.798	0.385
Italy	Fluoro-edenite	9.760	8.492	0.138	0.011	1.521	0.597
Russia	Anthophyllite	22.969	6.714	0.094	0.009	0.110	0.472
Australia	Crocidolite	18.998	0.361	0.109	0.000	0.686	0.025
Devon, England HSE	Actinolite	12.147	0.990	0.126	0.001	1.113	0.070
Wales	Crocidolite	6.154	5.492	0.128	0.007	2.732	0.385
Bolivia	Crocidolite	9.308	0.636	0.116	0.001	1.321	0.045
Yamaga Mine, Japan	Tremolite	9.041	4.606	0.112	0.006	1.268	0.324
Jamestown, CA	Tremolite	9.043	5.075	0.095	0.007	0.562	0.357
Korea	Tremolite	9.871	3.526	0.095	0.005	0.554	0.248
Oregon, ND	Erionite	13.115	0.995	0.101	0.001	1.160	0.070
Karain, Turkey	Erionite	11.431	2.608	0.085	0.003	0.604	0.183
South Africa	Crocidolite	20.667	0.644	0.108	0.001	1.075	0.045
HSE	Actinolite	9.602	3.081	0.095	0.004	0.609	0.216
HSE	Tremolite	7.465	2.515	0.095	0.003	0.720	0.177
HSE Sample 2	Tremolite	7.709	4.606	0.130	0.006	1.900	0.324
Metsovo, Greece	Tremolite	9.560	4.357	0.097	0.006	0.758	0.306
Québec, Canada	Chrysotile	17.065	0.723	0.109	0.001	1.667	0.051
Calidria, CA	Chrysotile	9.082	2.659	0.089	0.004	1.843	0.187
Swift Creek, WA	Chrysotile	8.100	4.606	0.129	0.006	5.599	0.324
Balangero, Italy	Chrysotile	9.419	4.747	0.079	0.006	1.555	0.334
Armley, England	Chrysotile	11.461	3.655	0.063	0.005	0.323	0.257
Eastern New York	Tremolite	11.913	2.472	0.084	0.003	0.434	0.174
Gouverneur, NY	Talc	11.050	4.357	0.098	0.006	0.010	0.306
Synthetic (citation)	Na-clinojimthompsonite	5.010	18.990	0.090	0.025	1.088	1.335
El Dorado Hills, CA	Tremolite	6.964	1.363	0.128	0.002	2.151	0.096
Calaveras Dam, CA	Glaucophane	9.020	18.990	0.110	0.025	0.388	1.335
Libby, MT	Na-Ca amphibole	8.407	1.899	0.124	0.003	1.619	0.133
Boulder City, NV	Actinolite	7.929	2.412	0.122	0.003	1.568	0.169
Rock Hill Quarry, PA	Actinolite	8.796	2.659	0.111	0.004	0.942	0.187
Balangero, Italy	Balangeroite	18.448	10.964	0.133	0.015	3.912	0.770
Italy	Fluoro-edenite	7.792	10.964	0.138	0.015	1.309	0.770
Marbridge mine Québec, Canada	Actinolite	7.491	18.990	0.144	0.025	1.375	1.335
Afghanistan	Anthophyllite	5.288	18.990	0.128	0.025	1.815	1.335
NIOSH	Tremolite	10.800	18.990	0.119	0.025	0.340	1.335
Barstow, CA	Tremolite	8.363	1.698	0.130	0.002	1.761	0.119

**TABLE 4:** Average dimensional characteristics of EMPs with L > 5 µm and W > 0.15 µm.

Location	Mineral type	Length (µm)		Width (µm)		Rigidity index (µm^2^ x GPa x 10^4^)	
		Mean	StDev	Mean	StDev	Mean	StDev
Shadwell Quarry, VA	Actinolite	8.58	1.4	1.52	0.04	25415.07	1,262.1
Boulder City, NV	Actinolite	10.46	0.55	1.02	0.02	1,159.72	492.27
Fairfax, VA	Actinolite	8.71	1.01	1.07	0.03	1,319.93	913.58
San Bernardino, CA	Actinolite	7.85	1.04	1.63	0.03	37647.26	942.63
Enoree, SC	Actinolite	9.74	3.17	0.52	0.09	891.81	2,857.21
Rock Hill Quarry, PA	Actinolite	8.77	1.59	0.71	0.05	616.6	1,434.22
Marbridge Mine Québec, Canada	Actinolite	7.38	0.9	1.43	0.03	23808.15	813.24
Devon, England	Actinolite	18.83	0.74	0.24	0.02	15.61	671.7
Great Britain	Actinolite	6.79	1.24	1.2	0.04	18993.91	1,115.34
Great Britain	Actinolite	9.87	1.32	0.38	0.04	74.64	1,191.54
Transvaal, SA	Amosite	16.99	0.25	0.49	0.01	151.72	228.74
India	Anthophyllite	9.43	1.03	1.21	0.03	1838.39	931.61
Sweden	Anthophyllite	7.86	2.13	1.48	0.06	27252.6	1918.17
Finland	Anthophyllite	11.33	0.82	0.68	0.02	428.91	742.38
Russia	Anthophyllite	11.32	3.22	0.51	0.09	161.31	2,902.92
Montauban mine Québec, Canada	Anthophyllite	7.83	1.29	1.28	0.04	22319.96	1,163.42
Sal Mountain, ID	Anthophyllite	9.2	1.25	0.75	0.04	602.03	1,126.12
Czechoslovakia	Anthophyllite	9.23	1.23	1.13	0.04	9,612.34	1,107.46
Afghanistan	Anthophyllite	8.55	1.3	1.51	0.04	24590.4	1,169.5
Balangero, Italy	Balangeroite	12.84	2.46	0.68	0.07	14607.74	2,220.13
Québec, Canada	Chrysotile	13.78	0.38	0.47	0.01	3,024.38	344.51
Calidria, CA	Chrysotile	13.36	3.51	0.46	0.1	1,483.62	3,169.79
Swift Creek,WA	Chrysotile	9.09	3.91	0.76	0.11	27373.63	3,527.01
Balangero, Italy	Chrysotile	9.67	12.67	0.57	0.37	1960.28	11428.83
Armley, England	Chrysotile	14.2	12.67	0.29	0.37	195.05	11428.83
Australia	Crocidolite	25.78	0.54	0.27	0.02	23	484.47
Wales sample	Crocidolite	8.63	1.28	0.45	0.04	129.1	1,154.49
Bolivia	Crocidolite	11.57	0.26	0.48	0.01	253.25	238.36
South Africa	Crocidolite	16.79	0.48	0.39	0.01	93.68	436.83
Rome, OR	Erionite	12.97	0.48	0.55	0.01	5,850.57	432.28
Karain, Turkey	Erionite	13.96	2.05	0.56	0.06	5,611.08	1854
South Africa	Ferro-actinolite	9.98	1.42	0.46	0.04	116.86	1,277.78
New York	Fibrous talc	13.35	1.92	0.58	0.06	28.76	1732.83
Italy	Fluoro-edenite	17.04	1.09	1.03	0.03	4,038.84	983.64
Italy	Fluoro-edenite	18.33	1.76	0.5	0.05	68.7	1,584.89
Calaveras Dam, CA	Glaucophane	9.05	0.2	0.86	0.01	1,193.45	179.09
Homestake, SD	Grunerite	7.65	0.59	1.48	0.02	33758.62	528.58
Portugal	Grunerite	7.05	3.12	1.43	0.09	30751.64	2,813.58
Labrador, Canada	Grunerite	7.52	0.9	1.39	0.03	26756.83	815.31
Finland	Grunerite	7.57	0.4	1.48	0.01	33594.63	358.11
Taconite mine, MN	Grunerite/actinolite	7.55	2.87	1.6	0.08	36556.91	2,588.12
Goonyella Mine, Australia	Hornblende	20.28	1.36	1.33	0.04	680.37	1,225.3
Libby, MT	Na-Ca amphibole	10.79	0.3	0.72	0.01	548.62	272.7
Long Valley Creek, CA	Riebeckite	16.94	1.05	1.25	0.03	12632.44	944.24
St Peter’s Dome, CO	Riebeckite	6.99	1.25	1.5	0.04	34927.67	1,131.62
Pilbara, Australia	Riebeckite	7.58	0.25	0.89	0.01	7,261.61	228.37
Colorado (CO)	Riebeckite	7.21	0.89	1.34	0.03	27449.06	805.13
Pikes Peak, CO	Riebeckite	8.39	1.08	1.81	0.03	48341.92	978.22
El Dorado Hills, CA	Tremolite	9.38	0.46	1.44	0.01	2,568.79	418.3
Udaipur, India	Tremolite	7.75	2.99	0.79	0.09	852.71	2,693.8
Lone Pine, CA	Tremolite	11.87	0.64	0.36	0.02	61.25	580.21
Miners Bay, Canada	Tremolite	8.42	5.4	0.84	0.16	2,353.47	4,873.27
Barstow, CA	Tremolite	11.92	0.47	0.74	0.01	7,426.76	424.9
Gouverneur, NY	Tremolite	7.68	2.87	1.62	0.08	33278.58	2,588.12
Brazil	Tremolite	7.45	0.72	1.31	0.02	22692.18	651.75
Sparta, NJ	Tremolite	7.79	1.27	1.07	0.04	9,479.14	1,142.88
Madagascar	Tremolite	8.06	0.63	1.31	0.02	20545.38	564.09
Yamaga Mine, Japan	Tremolite	14.31	1.29	0.55	0.04	265.5	1,166.45
Shinness, Scotland	Tremolite	7.71	0.91	1.32	0.03	21693.41	818.44
Dornie, GB	Tremolite	7.39	1.31	1.56	0.04	33710.81	1,181.94
Ala di Stura, Italy	Tremolite	9.24	1.29	1.22	0.04	2,328.5	1,166.45
Swansea Lab	Tremolite	10.16	1.24	1.15	0.04	12926.44	1,115.34
Jamestown, CA	Tremolite	8.68	1.26	0.74	0.04	857.35	1,137.21
Korea	Tremolite	11.67	1.38	0.39	0.04	164.2	1,246.99
Canada	Tremolite	8.99	1.18	1.4	0.03	21889.66	1,061.14
HSE	Tremolite	10.57	1.44	0.38	0.04	132.94	1,298.23
HSE	Tremolite	11.66	1.3	0.37	0.04	115.97	1,175.67
Metsovo, Greece	Tremolite	8.25	2.73	0.41	0.08	384.52	2,464.8
Falls Village, CT	Tremolite	7.55	4.22	1.1	0.12	15745.26	3,809.61
Eastern New York	Tremolite	16.95	3.17	0.5	0.09	541.54	2,857.21
Québec, Canada	Tremolite	7.47	5.66	0.96	0.16	5,760.92	5,111.13
NIOSH sample	Wollastonite	10.65	1.14	1.71	0.03	26833.07	1,024.28
Synthetic	Na-clinojimthompsonite	7.03	6.77	0.32	0.2	46.81	6,108.96

Based on human epidemiological data, we have found that the proportion of EMPA is a measure of the potency of the dust overall (Wylie et al., 2020; Korchevskiy and Wylie, 2021; Wylie and Korchevskiy, 2023). It provides a population measure of an aerosol that is highly correlated with mesothelioma risk from its inhalation. Table 5 provides the proportion of EMPA in total EMP with L > 5 µm.

**TABLE 5:** Proportion of EMPA among all EMP (L > 5 µm).

Location	Mineral type	Habit	Fraction EMPA
Shadwell Quarry, VA	Actinolite	NA	0.00
Boulder City, NV	Actinolite	M	0.05
Fairfax, VA	Actinolite	A	0.00
San Bernardino, CA	Actinolite	NA	0.00
Enoree, SC	Actinolite	NA	0.00
Rock Hill Quarry, PA	Actinolite	M	0.29
Marbridge mine Québec, Canada	Actinolite	NA	0.00
Devon, England HSE sample	Actinolite	A	0.39
ROM sample	Actinolite	NA	0.00
HSE	Actinolite	A	0.17
Transvaal, SA	Amosite	A	0.06
India	Anthophyllite	A	0.02
Sweden	Anthophyllite	NA	0.00
Finland	Anthophyllite	A	0.02
Russia	Anthophyllite	A	0.21
Montauban mine Québec, Canada	Anthophyllite	NA	0.00
Salz Mountain, GA	Anthophyllite	A	0.00
Czechoslovakia	Anthophyllite	M	0.00
Afghanistan	Anthophyllite	NA	0.01
Balangero, Italy	Balangeroite	M	0.05
Québec, Canada	Chrysotile	A	0.24
Calidria, CA	Chrysotile	A	0.66
Swift Creek, WA	Chrysotile	A	0.45
Balangero, Italy	Chrysotile	A	0.89
Armley, England	Chrysotile	A	0.93
Australia	Crocidolite	A	0.71
Wales sample	Crocidolite	A	0.06
Bolivia	Crocidolite	A	0.16
South Africa	Crocidolite	A	0.39
Oregon, ND	Erionite	A	0.21
Karain, Turkey	Erionite	A	0.41
Transvaal Mountain leather	Ferro-actinolite	A	0.03
Gouverneur, NY	Talc	A	0.18
Italy	Fluoro-edenite	NA	0.01
Italy	Fluoro-edenite	A	0.05
Calaveras Dam, CA	Glaucophane	M	0.02
Homestake, SD	Grunerite	NA	0.00
Portugal	Grunerite	NA	0.00
Labrador, Canada	Grunerite	NA	0.00
Finland	Grunerite	NA	0.00
Chatfield’s sample	Grunerite	NA	0.00
Taconite mine, MN	Grunerite and actinolite	NA	0.00
Goonyella Mine, Australia	Hornblende	A	0.00
Libby, MT	Libby amphiboles	M	0.03
Long Valley Creek, CA	Riebeckite	NA	0.00
St Peter’s Dome, CO	Riebeckite	NA	0.00
Pilbara, Australia	Riebeckite	NA	0.00
Chatfield’s sample	Riebeckite	NA	0.00
Pikes Peak, CO	Riebeckite	NA	0.00
El Dorado Hills, CA	Tremolite	M	0.00
Udaipur, India	Tremolite	A	0.03
Lone Pine, CA	Tremolite	A	0.09
Miners Bay, Canada	Tremolite	NA	0.00
Barstow, CA	Tremolite	M	0.08
Gouverneur, NY	Tremolite	NA	0.00
Brazil	Tremolite	NA	0.00
Sparta, NJ	Tremolite	NA	0.00
Madagascar	Tremolite	NA	0.00
Yamaga Mine, Japan	Tremolite	A	0.08
Shinness, Scotland	Tremolite	NA	0.00
Dornie, GB	Tremolite	NA	0.00
Ala di Stura, Italy	Tremolite	M	0.00
Swansea Lab	Tremolite	NA	0.00
Jamestown, CA	Tremolite	A	0.06
Korea	Tremolite	A	0.15
NIOSH cleavage fragments	Tremolite	NA	0.00
HSE	Tremolite	A	0.27
HSE	Tremolite	A	0.08
Metsovo, Greece	Tremolite	A	0.31
Falls Village, CT	Tremolite	NA	0.00
Eastern New York	Tremolite	A	0.65
Québec, Canada	Tremolite	NA	0.00
NIOSH sample	Wollastonite	NA	0.00
Synthetic	Na clinojimthompsonite	A	0.13

A, asbestiform; NA, non-asbestiform; M, mixed habit.

Researchers working with silicates have not placed a lower limit of width in defining mesotheliomagenic fiber. Yet it seems clear that exposures to many MWCNT populations that are very narrow do not result in mesothelioma in animals, and there may be a low limit of width for mesotheliomagenic silicate fiber as well.

### 3.5 Measures of rigidity

Stiffness is the resistance to bending and can be modeled according to the Euler buckling theory, which gives the critical threshold force necessary to buckle an elongate elastic particle when a force is applied to the particle’s end.

The critical buckling force F_crit_ is given by the following formula:

$$F_{\text{crit}}=\pi^{2}{\text{EI}}_{A}/L_{\text{eff}}^{2}$$ (1)

Where E = Young’s modulus parallel to elongation and I_A_ is the area moment of inertia. EI_A_ is also referred to as the bending stiffness or flexural rigidity. Flexural rigidity is independent of length.

There are four Euler buckling modes. For the first, which assumes one end of the fiber is attached and the other free, L_eff_ = 2L and Equation 1 becomes:

$$F_{\text{crit}1}=\pi^{2}{\text{EI}}_{A}/4L^{2}$$ (2)

For the second buckling mode, which assumes both ends of the fiber are fixed, Equation 1 becomes:

$$F_{\text{crit}2}=\pi^{2}{\text{EI}}_{A}/L^{2}$$ (3)

In his work on bending and lysosomal disruption, Zhu et al. (Zhu et al., 2016) assume the second buckling mode and F_crit_ is referred to as the Critical Buckling Load or Critical Buckling Force. A case could also be made for using the first mode as might occur if a fiber pierces a cell wall. Because of the uncertainty of the mode, among other things, and because we want to compare population characteristics, we calculate a **Rigidity Index** (RI) by using the convention RI = F_crit1_ x 10^4^:

$$Rigidity\;Index\;\left(\text{RI}\right)=\pi^{2}{\text{EI}}_{A}/4L^{2}\;x\;10000$$ (4)

Because we have both length and width measurements in our data sets, the RI can be calculated for every particle in a set and averaged for the population characteristic.

The shape of the cross section of the elongated particle (I_A_) is an important variable in RI:

$$\text{For }a\text{ circular cross section},I_{A}=\pi \;r^{4}/4\text{ where }r=½\;W;$$ (5)

$$\text{For }a\text{ square cross section},I_{A}=W^{4}/12$$ (6)

$$\text{For }a\text{ rectangular cross section},I_{A}=\left(T^{3}\right)\left(W\right)/12\text{ where }T=\text{thickness}.$$ (7)

For a hollow tube where W_0_ is the outer diameter and W_i_ is the inner diameter:

$$I_{A}=\pi \;\left(W_{o}^{4}-W_{i}^{4}\right)\;/\;64$$ (8)

Broβell et al. (2020) applied the Euler buckling formula in an analysis of the buckling of an elongate particle by a macrophage to determine flexural rigidity. They concluded, assuming the conditions of flexural rigidity (slightly modified Equation 1) that if the rigidity index of the fiber equals or is less than approximately 10^−19^ N × m^2^ then a force of approximately 10 nN exerted by a macrophage will be able to bend the fiber, facilitating its removal. Below a **critical diameter** for each fiber type, a fiber would lose biological rigidity and that critical diameter is controlled by the Young’s modulus (E) parallel to elongation. Their estimates are:

$$\text{MWCNT }\left(E=360-100\text{ GPa}\right)\text{ Critical Diameter}=37-44\text{ nm}$$

$$\text{Chrysotile }\left(E=160\text{ GPa}\right)\text{ Critical Diameter}=60\text{ nm}$$

$$\text{Crocidolite }\left(E=190\text{ GPa}\right)\text{ Critical Diameter}=56\text{ nm}.$$

Chrysotile fibrils are cylindrical tubes with exterior and interior diameters that vary slightly across occurrences. Chisholm et al. (1983) measured the outer diameter from 15 locations and the inner diameter from 7 of these. Mean outer diameters ranged from 13 to 38 nm with individual fibril outer diameters ranging from 10 to 85 nm. Inner diameters range from 0 to 10 nm. Because of the relatively small size of the inner tube diameter, it can be ignored in the calculation of the rigidity index, as it serves to lower RI only slightly.

The mean length of chrysotile fibrils depends on location. Widths larger than about 40 nm mean that the fiber is composite. Atkinson et al. (1970) measured lengths of single fibrils and estimate mean lengths of <2 and 4 μm, but report single fibril lengths up to several millimeters or more, although they caution that the longest fibrils may be fragile.

According to the study of Broβell et al. (2020), a 60 nm upper limit for biological rigidity would preclude almost all single chrysotile fibrils. Measurements of chrysotile populations from aerosols or bulk samples are often made on composite fibers so the calculated rigidity would be greater than if they were single fibrils. Their calculated rigidity would be higher. However, chrysotile fibrils may disaggregate following inhalation into these biologically weak single fibrils. The degree of disaggregation may vary among occurrences depending on interfibrillar adhesion.

A major uncertainty in applying the Euler buckling theory comes from the importance of thickness in determining I_A_ for particles with a rectangular cross section, as Equation 7 shows. Wylie et al. (1982) measured the thickness and determined a relationship between log thickness and log width for crocidolite and amosite that can be expressed as follows:

Log thickness=0.692⁡log⁡width– 0.493 (9)

This relationship was used in this study to estimate thickness from measured width for all asbestiform amphiboles. For nonasbestiform amphiboles, the geometric shape produced by the regular cleavage in two directions at an angle of 56° is a rhombus, and this shape results in a width to thickness ratio (W:T) of 1.88. Erionite, balangeroite, and talc belong to silicate structural groups that are different from amphiboles, precluding the application of the amphibole models. Erionite is a framework silicate with an open structure, balangeroite is a single chain silicate, and talc is a sheet silicate. Optical observations for erionite, balangeroite, and talc were used to approximate a W:T for each as follows: 1:1 for erionite, 1.1:1 for balangeroite, and 3:1 for talc. There are no published measurements of these ratios.

Young’s modulus is a measure of lattice strength, and should not vary with habit. It will vary with lattice direction, however. For EMPs, Young’s modulus is normally given for the long direction only. It has been measured for a variety of silicates, including all of the major types of asbestos. Based on a literature review, we list average values of Young’s modulus measured directly on minerals of interest to lung pathology in Table 6.

**TABLE 6:** Young’s modulus in GPa for minerals.

Mineral	GPa	Source
Crocidolite and Riebeckite	180	(Aveston, 1969) (Golden, 1968)
Actinolite	150	Golden (1968)
Ferro-actinolite	164	Aveston (1969)
Amosite and Grunerite	180	(Aveston, 1969) (Golden, 1968)
Anthophyllite	158	Aveston (1969)
Tremolite	165	(Aveston, 1969) (Golden, 1968)
Other amphiboles (glaucophane, fluoro-edenite)	165	Estimate
Chrysotile	170	(Aveston, 1969) (Golden, 1968)
Balangeroite	165	Estimate
Erionite	45	Bryukhanov et al. (2014)
Talc	16	Broz et al. (2006)
K-feldspar	100	Liu et al. (2023)
Illite	5–67	Hulan et al. (2020)
Kaolinite	40	Elhachemi et al. (2024)
Biotite	45	Liu et al. (2023)

In the cases in which there are no direct measurements, we made the following assumptions to provide the data in Table 6. For erionite we used published data on zeolites which were very similar for different types. For balangeroite, we assumed an amphibole average given that the published Young’s moduli for other single chain silicates were very similar to the amphiboles as a group. Amphiboles without direct measurement of Young’s modulus were assigned 165 GPa.

### 3.6 Calculation of the rigidity index

Using the Euler formulae, we calculated RI for the MWCNT populations (Table 1) and for three sets of EMP populations by location (Tables 2–4). RI was determined for each particle independently and then averaged.

## 4 Results

### 4.1 MWCNT: average length, width, and outcome

**FIGURE 1:**
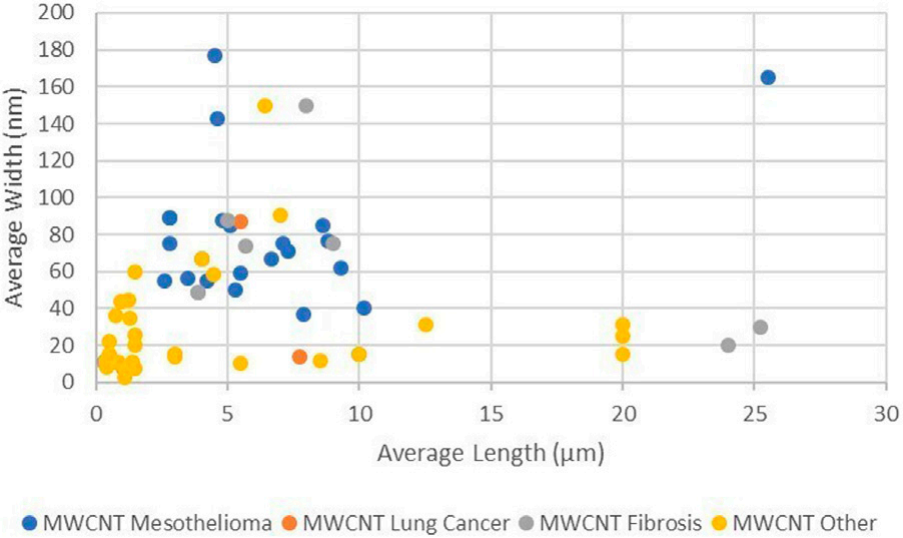
Average length and width of MWCNT by outcome (log scales).

Figure 1 shows the distribution of average length and average width of the MWCNT by reported disease outcome. While there is clustering of the mesothelioma, lung cancer, and fibrosis outcomes, within that cluster there are populations with similar lengths and widths that did not result in these diseases. Differences in outcome are expected where the dose, duration of dose, method of dose administration, length of time from dose to autopsy, and type of animal are variables. A more detailed study of the negatives within this cluster would be helpful in evaluating other characteristics of the populations that affect outcome.

Figure 1 shows that the average lengths of all reported carcinogenic and fibrogenic populations are greater than 2 µm. These populations will contain fibers that are longer than 5 µm. There are fiber populations with average length greater than 2 µm as well producing no carcinogenic outcome, so average length is not necessarily the sole determinant of outcome.

Figure 1 also demonstrates that there are no fiber populations with average width narrower than 37 µm producing mesothelioma or lung cancer in animals. This is the same minimum diameter of CNTs predicted to have biological rigidity (Zhu et al., 2016) in the animal model, it appears that MWCNT fiber populations can be too narrow or too short to be carcinogenic.

### 4.2 MWCNT: average length, width, rigidity, and outcome

**FIGURE 2:**
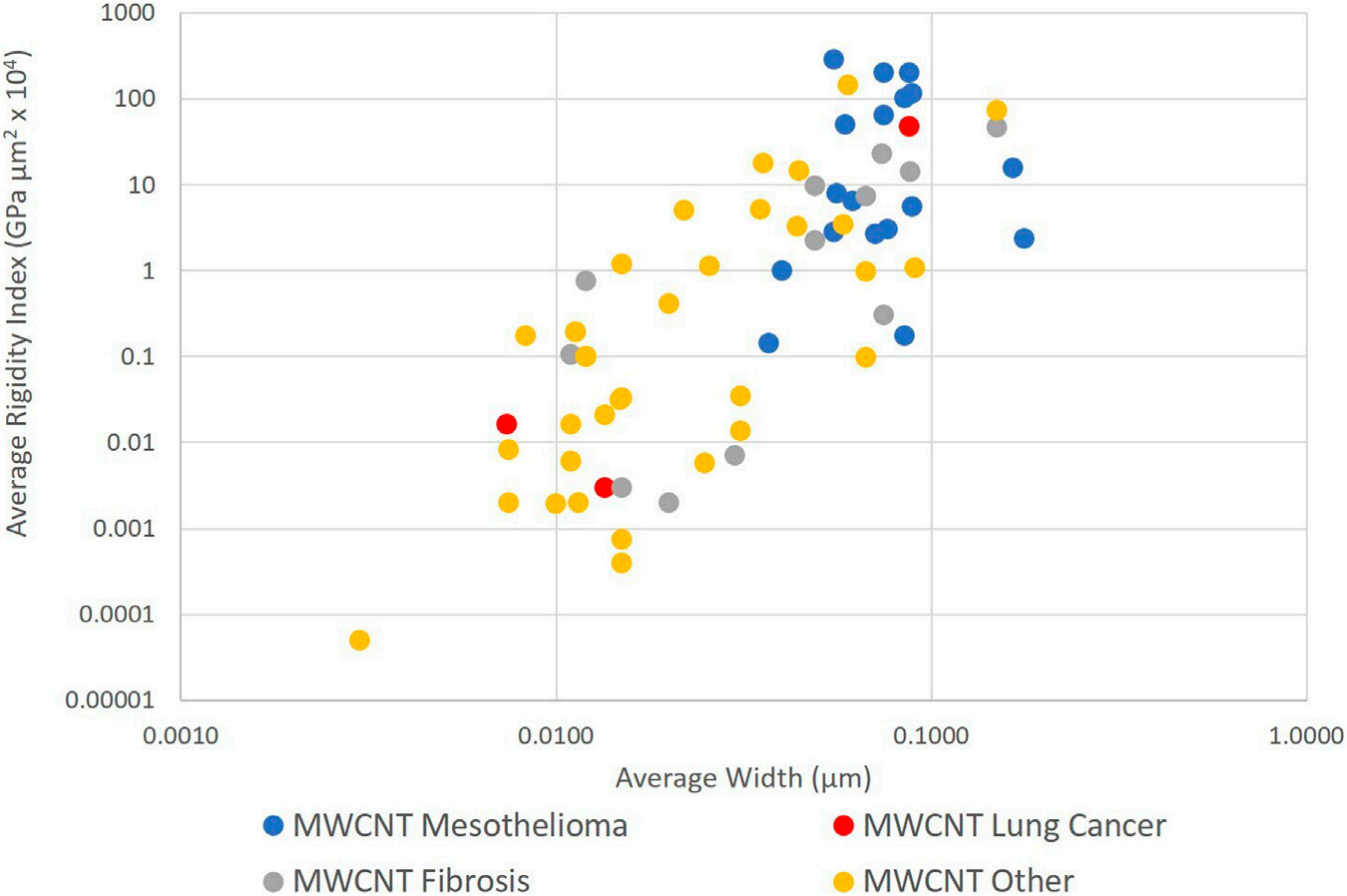
Average length, width, and rigidity of MWCNT by outcome (log scales).

Figure 2 shows that when width and rigidity are considered together, MWCNT group by disease outcome. First, a high rigidity separates those MWCNT that have been shown to produce mesothelioma in animals, from effectively all other MWCNT exposures. A level of RI between 0.1 and about 0.02 separate mesothelioma from other outcomes. For purposes of this paper, a RI of 0.05 (µm^2^ x GPa x 10^4^) was selected as an estimate of the central value for the lower limit of rigidity that limits mesotheliomagenic populations for modelling purposes. This is equivalent to a bending force of 2.94 nN.

The smallest average width among the MWCNT causing mesothelioma is 37 nm, consistent with the estimates of Broβell et al. (2020) for biologically weak MWCNT. This minimum width separates MWCNT populations that result in both lung cancer and mesothelioma. The smallest average length for a mesotheliomagenic MWCNT is about 2 µm.

### 4.3 EMP: average length, width, and rigidity

**FIGURE 3:**
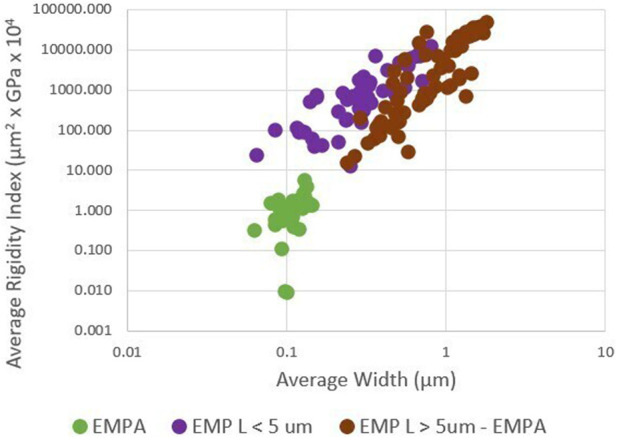
Rigidity index and EMP groups (log scales).

Figure 3 shows that the three EMP groups can be isolated based on average length, width, and rigidity index. These three groups were selected for the following reasons. EMPA is found in exposures to asbestiform minerals associated with elevated mesothelioma (Wylie et al., 2020). EMPA is not found in exposures to fragmented amphibole so EMP L > 5 – EMPA approximates fragmented amphibole and asbestiform amphibole particles not associated with significant risk for mesothelioma. EMP L < 5 cannot be used for quantitative risk assessment.

Most elongate silicate particles exceed a threshold rigidity of 0.05. This validates the assumption that silicate minerals generally behave as rigid solids when they form as elongate mineral particles.

**FIGURE 4:**
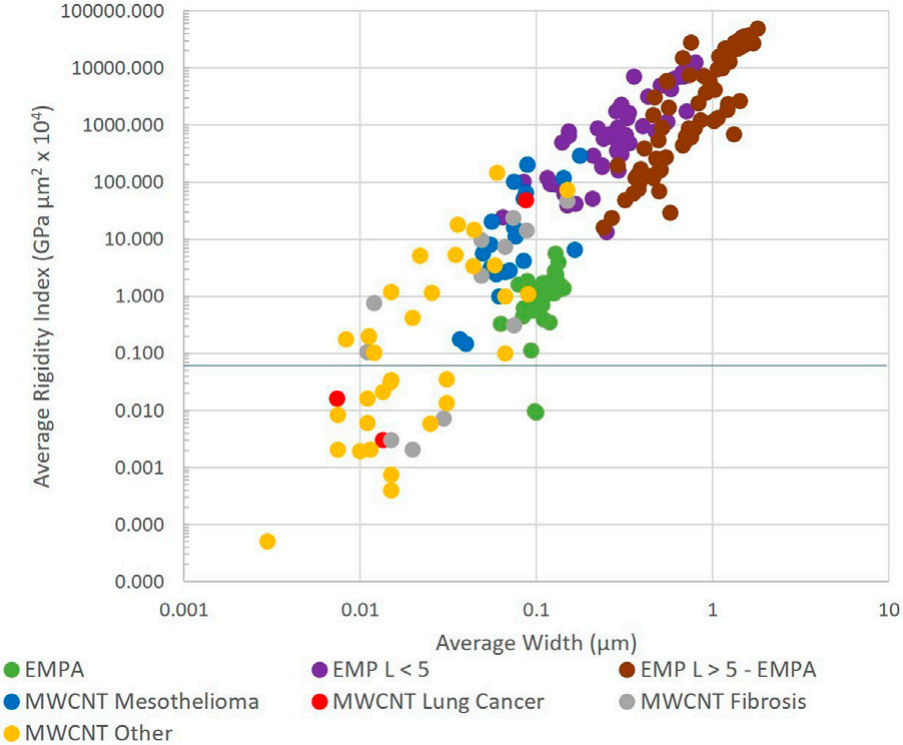
Average width and average rigidity index of multiwalled carbon nanotubes (MWCNT) and elongated mineral particles (EMP) (log scales).

Figure 4 shows the average width and average rigidity index of both EMP and MWCNTs.

### 4.4 EMP: rigidity and individual fiber sizes

Based on the Euler model (Equation 4) and an assumed square cross section (Equation 7), we can calculate the rigidity index for minerals with different Young’s modulus and dimensional parameters for comparison. Chrysotile, amphibole, and balangeroite have very similar values of Young’s modulus, about 165 GPa. An EMP with a Young’s modulus of 165 GPa and a width and thickness of 0.06 µm would have RI < 0.05 (µm^2^ x GPa x 10^4^) when length exceeds about 20 µm. With a width and thickness of 0.1 µm, RI < 0.05 (µm^2^ x GPa x 10^4^) occurs when length exceeds 52 µm. With a width and thickness of 0.15 µm, the RI of EMPs would remain >0.05 (µm^2^ x GPa x 10^4^) until about 115 µm in length.

For the same Young’s modulus, if the cross section were rectangular with a width to thickness ratio of 3:1, a fiber with a measured width of 0.06 µm would have RI < 0.05 (µm^2^ x GPa x 10^4^) when length exceeds ~ 3.5 µm. The same cross section of a ribbon shaped fiber with measured width of 0.15 µm would produce a RI < 0.05 (µm^2^ x GPa x 10^4^) for fibers longer than about 22 µm.

If the Young’s modulus were 16.5 in instead of 165 GPa, for example, for talc, a fiber with a square cross-section and width and thickness of 0.06 µm would have an RI < 0.05 (µm^2^ x GPa x 10^4^) above a length of about 6 µm. If its width were 0.15 µm*,* the RI of fibers would exceed 0.05 when they exceed about 37 µm in length. If width were three times thickness, a fiber with measured width of 0.15 would have a RI < 0.05 (µm^2^ x GPa x 10^4^) when length exceeds about 7 µm.

### 4.5 EMP: low rigidity fiber

The mineral populations that contain fibers with low rigidity are restricted to the asbestiform habit. Long cleavage fragments are not narrow enough and the narrowest fragments are not long enough to affect rigidity in a biologically significant way.

Table 7 lists the proportion of EMPA fibers that have a rigidity index below 0.05 by mineral (combining locations from Tables 2–4) and habit. Also shown is the abundance of fiber with RI below 0.01 for comparison.

**TABLE 7:** Proportions of EMPA low rigidity fibers for various minerals.

Mineral type	Habit	RI (µm^2^ x GPa x 10^4^) Mean	RI (µm^2^ x GPa x 10^4^) StDev	Fraction of fibers with RI < 0.05 (µm^2^ x GPa x 10^4^)	Fraction of fibers with RI < 0.01 (µm^2^ x GPa x 10^4^)
Chrysotile	A	1.69	0.03	0.08	0.01
Cummingtonite-grunerite	A	0.56	0.03	0.16	0.02
Cummingtonite-grunerite	NA	1.44	1.42	0.00	0.00
Riebeckite	A	0.39	1.42	0.12	0.03
Riebeckite	NA	0.90	0.06	0.00	0.00
Actinolite	A	1.37	1.42	0.03	0.00
Actinolite	NA	1.28	0.13	0.00	0.00
Anthophyllite	A	0.54	0.15	0.14	0.01
Anthophyllite	NA	1.81	1.42	0.00	0.00
Tremolite	A	0.94	0.05	0.05	0.01
Tremolite	NA	0.98	1.01	0.00	0.00
Glaucophane	M	2.12	0.12	0.00	0.00
Na-Ca amphibole	M	1.63	0.16	0.00	0.00
Fluoro-edenite	A	1.57	0.43	0.08	0.00
Fluoro-edenite	NA	1.31	0.82	0.00	0.00
Erionite	A	1.15	0.07	0.11	0.05
Balangeroite	A	3.91	0.82	0.00	0.00
Talc	A	0.01	0.33	1.00	0.74
Syn Na-clinojimthompsonite	A	1.09	1.33	0.00	0.00

A, asbestiform; NA, non-asbestiform; M, mixed habit.

### 4.6 Rigidity by silicate mineral group

Silicate minerals make up more than 90% of rock on the surface of the earth. They are classified according to the polymerization of their major structural element, the Si-O tetrahedron. Mineral fiber could occur within any of the structural groups, but in issues surrounding inhalation toxicology and occupational health, the four most common are the inosilicates (chain silicates including amphiboles and balangeroite), phyllosilicates (sheet silicates including chrysotile, palygorskite, sepiolite and talc), and tectosilicates (framework silicates including the zeolite group (erionite). A few members of the nesosilicates (island silicates including the alumino-silicates) also form EMPs.

Silicate minerals have values of Young’s modulus that are characteristic of their silicate structure. For example, the GPa values for all amphibole minerals are surprisingly similar because a double-chain silicate is the strongest structural unit common to them all. The other cations that are present will influence Young’s modulus, but by no more than about 10% within the group average. Similarly, other silicate structure types generally will have characteristic ranges of Young’s modulus values.

#### 4.6.1 Inosilicates

Chain silicates include all EMPA minerals listed in Tables 2–4 except the sheet silicates chrysotile and talc and the framework silicate erionite. Chain silicates are structurally strong and only a small portion of EMPA from asbestiform chain silicates have RI < 0.05. Given the same cross-sectional shape, and fairly uniform widths (Table 3), length can be a major factor driving their average rigidity. For example, the high proportion of tremolite fibers from Udiapur, India with low RI can be attributed to its long length, and the higher RI for the crocidolite from Wales can be attributed to its shorter length.

#### 4.6.2 Phyllosilicates

The phyllosilicates silicates can be divided into four groups based on structure and composition: 1) serpentine, 2) mica, 3) chlorite, and 4) clay.

Chrysotile is a member of the serpentine group. Its fibrils are tubes formed from a rolled silicate sheet. This form is mechanically very strong and gives chrysotile a Young’s modulus that is equivalent to the chain structures. Chrysotile is only one of a group of phyllosilicate nano-scrolls, which have a reported range in Young’s modulus of 150–300 GPa (Krusilin et al., 2022). Despite the high strength, nano-scrolls with the width of single chrysotile fibrils (20–40 nm) will lack rigidity above 5 µm in length.

There are reports of a few occurrences of the fibrous forms of minerals in the chlorite and mica groups. In both, there are weak ionic bonds between the silicate sheets. Young’s modulus for biotite was measured as 45 GPa (Liu et al., 2023).

For minerals in the clay group, there are only van de Waals forces holding the silicate sheets together suggesting a very low Young’s modulus. It is not surprising that talc has a Young’s modulus of 16 GPa. Illite is a mineral with properties between clay and mica. In a study of five illite-bearing clays, Hulan et al. (Hulan et al., 2020) report a range in Young’s modulus from a low of less than 5 GPa to a high of about 67 GPa (possibly reflecting variable amounts of micaceous components).

Low Young’s modulus measurements would be expected for fibrous members of the clay group including **fibrous talc**, **palygorskite,** and **sepiolite**. Members of these groups would be expected to have a ribbon-like cross-section as we assumed for talc. Galan (Galan, 1996) reports width as 10–30 nm and thickness of 5–10 nm for palygorskite and sepiolite, confirming the ribbon-like cross section with w/t = 2–3. Wider fibers of palygorskite ranging from about 20 to 70 nm and 0.2–5,000 µm in length were reported by Tang et al. (Tang et al., 2017). Trigueiro et al. (Trigueiro et al., 2024) report width of 10–100 nm and length of 2–10 µm for sepiolite. The average width and length of talc fibers are given in Table 3. Given the low Young’s modulus for the nontubular clay phyllosilicates, their very narrow widths, and their ribbon-like form, most fibers from this group would not retain RI > 0.05 beyond several micrometers in length.

#### 4.6.3 Tectosilicates

The Young’s modulus for the most common framework silicates (quartz and feldspar) normally measures around 100 GPa. The zeolites, however, are characterized by an open structure and their Young’s modulus is about half that of feldspar.

#### 4.6.4 Nesosilicates

Generally, members of this silicate groups are not elongate. An exception is a group known as the alumino-silicates including kyanite, sillimanite, and andalusite. These minerals have high hardness and a structure containing chains of AlO_6_ octahedra, a strong structural element. For these reasons, they would be expected to have Young’s modulus similar to chain silicates.

### 4.7 EMP: dimension, biodurability, dose, and rigidity: risk for mesothelioma

The prevailing paradigm for mesotheliomagenic fiber is that dose, dimension, durability, and structural strength determine fiber potency. The rigidity index depends on the mechanical strength and the cross-sectional shape, as well as the width and length so it includes two parameters in the paradigm: mechanical strength and dimension, including assumptions about the third dimension, thickness. Durability can be represented by the biosolubility estimated by Gualtieri et al. (Gualtieri et al., 2018) as fiber lifetime determined by dissolution rates measured in simulated lung fluids.

Dimension is specified by the EMPA category, including the fraction of fibers longer than 5 μm that would also have width not higher than 0.15 μm (Wylie et al., 2020). The dimensions of particles also affect the rigidity index.

These data are given in Table 8. Also shown in Table 8 is the simple product of these three variables, and R_M_, the increase in mesothelioma expected for each fiber-year of exposure (Darnton, 2023).

**TABLE 8:** The data for modeling mesothelioma potency of various types of mineral fibers.

Mineral type	Habit	(a) EMPA	(b) RI (µm^2^ x GPa x 10^4)^	(c) Biosolubility (years)	Product: (a)*(b)*(c)	R_M_, %
Chrysotile	A	0.37	1.69	0.3	0.18828	0.0014*
Amosite	A	0.16	0.56	74	6.41333	0.11*
Grunerite	NA	0.00	1.44	74	0.02659	
Crocidolite	A	0.33	0.88	66	19.0103	0.52*
Riebeckite	NA	0.00	0.39	66	0.00404	
Actinolite	A	0.24	1.05	49	12.4467	
Actinolite	NA	0.00	1.37	49	0.01681	
Anthophyllite	A	0.04	0.54	245	5.84452	0.056**
Anthophyllite	NA	0.00	1.81	245	0.61573	
Tremolite	A	0.12	0.94	49	5.61877	
Tremolite	NA	0.00	0.98	49	0.02425	
Glaucophane	M	0.02	2.12	40	1.78663	0.0085***
Na-Ca amphiboles	M	0.03	1.63	49	2.13754	0.03*
Fluoro-edenite	A	0.02	1.57	140	4.17795	0.12***
Fluoro-edenite	NA	0.01	1.31	140	2.01399	
Erionite	A	0.18	1.15	181	36.9837	4.67***
Balangeroite	A	0.05	3.91	55.6	11.6509	0.045***
Talc	A	0.18	0.01	4.1	0.00705	

Sources of mesothelioma potency data: * (Darnton, 2023). **((Korchevskiy et al., 2013), based on Russian epidemiological data). *** Other sources: for erionite and fluoro-edenite, the potency was modeled and validated based on the epidemiological data (Korchevskiy et al., 2019). For glaucophane, the potency was modeled with dimensional characteristics and chemical composition (Korchevskiy et al., 2019). For balangeroite, the potency was modeled and validated by epidemiological data (Korchevskiy and Wylie, 2023). (A, asbestiform; NA, non-asbestiform; M, mixed habit).

The following regression equation can be derived between the Product variable and R_M_, with all mineral types with non-zero R_M_ from Table 8 included:

$$R_{M}=-0.54+0.12\text{ Product }\left(R=0.91,R^{2}=0.82,P<0.00071\right),$$ (10)

or alternatively

$$\begin{array}{c} {\mathrm{Log}}_{10}\left(R_{M}\right)=-2.09+1.38\log_{10}\left(\text{Product}\right)\\ \left(R=0.922,R^{2}=0.85,p<0.00040\right) \end{array}$$ (11)

If only Darnton (Darnton, 2023) R_M_ values from Table 8 would be included (for chrysotile, amosite, Libby amphiboles, and crocidolite), the correlation would be higher:

$$R_{M}=-0.03+0.02\text{ Product }\left(R=0.992,R^{2}=0.985,P<0.007\right),$$ (12)

or alternatively

$${\mathrm{Log}}_{10}\left(R_{M}\right)=-1.94+1.27\log_{10}\left(\text{Product}\right)\left(R=0.9996,R^{2}=0.999,p<0.00041\right).$$ (13)

if we would assume that all non-asbestiform mineral types would have R_M_ = 0 (%), the regression equation would be

$$R_{M}=-0.27+0.11\text{ Product }\left(R=0.895,R^{2}=0.8101,P<0.00001\right).$$ (14)

The non-linear regression equations appear to be more stable, with the coefficients not changing much with different sub-sets of the included mineral types (Darnton-only or all published).

Table 9 contains the results of the modeling of mesothelioma potency for various types and habits of mineral fibers based on EMPA, rigidity index, and biosolubility, based on Equation 13.

**TABLE 9:** Published and modelled values for mesothelioma potency factor R_M_ (%).

Mineral type	Habit	R_M_, % (published)	R_M_ (%), modeled by the product of EMPA, RI, and biosolubility
Chrysotile	A	0.0014	0.00138
Amosite	A	0.11	0.12162
Grunerite	NA	N/A	0.00011
Crocidolite	A	0.52	0.48341
Riebeckite	NA	N/A	0.00001
Actinolite	A	N/A	0.28231
Actinolite	NA	N/A	0.00006
Anthophyllite	A	0.056	0.10809
Anthophyllite	NA	N/A	0.00620
Tremolite	A	N/A	0.10281
Tremolite	NA	N/A	0.00010
Glaucophane	M	0.0085	0.02399
Na-Ca amphiboles	M	0.03	0.03013
Fluoro-edenite	A	0.12	0.07057
Fluoro-edenite	NA	N/A	0.02794
Erionite	A	4.67	1.12557
Balangeroite	A	0.045	0.25958
Talc	A	N/A	0.00002

A, asbestiform; NA, non-asbestiform; M, mixed habit.

**FIGURE 5:**
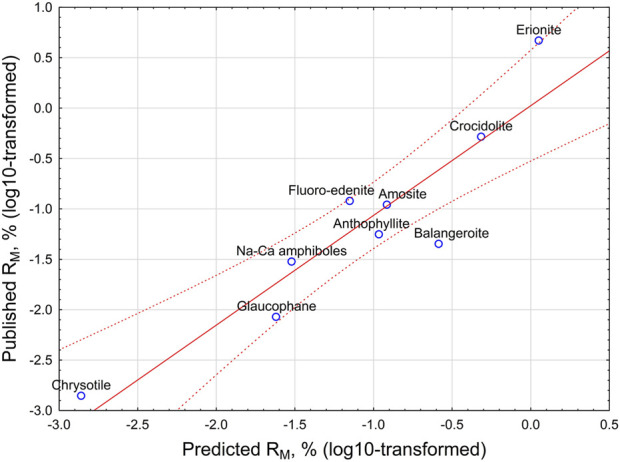
Relationship between predicted and published mesothelioma potency factor R_M_, % (log_10_-transformed). Straight line - linear regression equation. Dotted lines - 95% CI interval.

The correlation between the mesothelioma potency factor and the product of variables according to Equation 13 is illustrated in Figure 5.

As it can be seen from Table 9 and Figure 5, the model proposes quite precise estimations of the potency of chrysotile, amosite, Libby amphiboles, asbestiform anthophyllite, and crocidolite. Based on the model, erionite potency may be lower than reported. [See Stevens et al. (2024)], on the variability of possible potency estimations for erionite from various locations). Nevertheless, the model confirms erionite as the mineral with the highest mesothelioma potency.

The model would suggest higher potency estimations for glaucophane (0.02% vs previously estimated 0.0085%) and for balangeroite (0.26% vs 0.045%), both most probably because of higher rigidity index.

The potency for the non-asbestiform varieties of the minerals is much lower than for asbestiform analogies; for example, crocidolite is 48,000 times more potent than riebeckite fragments. The potency of fibrous talc is predicted as 0.00002%, lower than a reasonable level of statistical significance.

## 5 Discussion

Carcinogenicity of elongate mineral particles is a multi-dimensional chain of actions that includes different levels of interrelated steps (Kuroda, 2021). It is important for risk assessment purposes to elucidate quantitative predictors and boundary conditions for various characteristics of fibrous minerals that make them carcinogenic and distinguish them from other elongate mineral particles that are not carcinogenic.

The correlation between high rigidity and mesothelioma outcome in animal experiments with MWCNT makes a compelling case for the importance of rigidity in fiber carcinogenicity. When we included rigidity, biosolubility (Gualtieri et al., 2018) and EMPA, a simple model produced a high correlation between predicted and published R_M_ of mineral fiber, a measure of potency for mesothelioma (Wylie et al., 2020), as illustrated in Figure 5.

For most investigators who have experimented with mineral fiber, rigidity has been assumed. For the most part, this assumption is reasonable. It is only for mineral fiber that is less than about 60 nm in diameter such as single chrysotile tubes and the narrowest amphibole fibrils, or fiber with weak atomic structures and ribbon-like morphology typical of clay fiber, for which the lack of biological rigidity is likely a factor in reducing their mesotheliomagenic potential. This makes rigidity one of the vital boundary conditions that separates carcinogenic from non-carcinogenic particles.

Only fibers formed naturally can have the dimensions of EMPA; the narrowest might lack biological rigidity. Cleavage fragments are wider than fibers and do not form EMPA; cleavage fragments will always be rigid in biological systems.

EMPA is an important indicator for mesothelioma potency. Mineral dusts from locations where inhalation has resulted in excess mesothelioma contain EMPA. EMPA fibers possess many of the same characteristics as those MWCNT populations that produced mesothelioma in animals. Such MWCNT have RI between 0.1 and 100 and average widths <0.2 µm; EMPA fibers have W < 0.15 µm and RI from 0.01 to 10. Fibers with RI < 0.01 are not expected to be mesotheliomagenic.

Mesotheliomagenic EMP populations must contain rigid fibers with dimensions comparable to EMPA. Therefore in addition to rigidity, the dimensions of fibers create a second boundary condition for mesotheliomagenic particles.

Recently, a new metric of dimensions was introduced for both CNTs and EMPs: Dimensional Coefficient of Carcinogenicity (DCC) (Korchevskiy and Wylie, 2025). DCC depends on the relationship between surface area and a linear function of the third power of width for particles. It was demonstrated that for most mesotheliomagenic CNTs and amphibole elongate mineral particles, the average DCC is greater than 0.05. We can conclude that elongate particles, independent of their nature, should have DCC greater than 0.05 and RI > 0.05 to produce mesothelioma in humans.

The third boundary condition is the solubility of elongate particles, or, in a wider sense, their biopersistence (Laux et al., 2017). As we demonstrated, mesothelioma potency of mineral fibers can be modeled as a function of dimensions (EMPA), rigidity (RI for EMPA particles), and biosolubility (biopersistence), representing the three boundary conditions.

All single fibrils of chrysotile longer than a few micrometers will fall below the threshold for biological rigidity (RI = 0.05) because of their narrow widths. While it has generally been assumed that the low bioretention and carcinogenic potential of chrysotile is due to its high biosolubility, it may be that its low rigidity and ease of removal by macrophages are also responsible to some extent. Chrysotile fibers also likely break into shorter fibers following deposition in the lung and that would of course, change their rigidity and biopersistence. To the extent that bundles of several fibrils do not disaggregate, once they exceed about 60 nm in width, they will behave mechanically more like amphibole. The wider widths that characterize exposures in textile factories (Sebastien et al., 1989) may play a role in the higher incidence of mesothelioma observed there for this reason.

Minerals that belong to the sheet silicate group are known to form fibers. Direct measurement of thickness of minerals in this group is limited. However, because of their structure, mineral fibers from this group are likely to have a thickness that is significantly smaller than the measured width. The difference in width and thickness is probably greatest in the sheet silicate group referred to as clays which includes palygorskite, talc, and sepiolite, because they lack covalent or ionic bonding between the sheets. Because thickness is the smallest dimension, it effectively controls rigidity and must be known to establish sheet silicate carcinogenic potential. In addition, with the exception of those forming nanotubes (chrysotile, halloysite), Young’s modulus for this group is also generally less than 50 GPa.

What is the fate of the long, narrow, low rigidity EMPs once inhaled? They could “tangle”, increasing their effective aerodynamic diameter and promoting deposition before the alveolar space. Their lack of rigidity makes direct intercellular translocation unlikely. It might also enhance the probability that a macrophage would remove it, even though it is long, because its shape might be changed by the phagocytizing cell and it is unlikely to pierce membranes. Rigid fibers have aerodynamic diameters that are close to their actual diameters, and thin fibers despite being long, penetrate the alveolar region of the lung and can translocate to the pleura most readily. Further studies are needed to develop models of lung deposition for particles with different level of rigidity at various parts of human respiratory system, but it is obvious that rigidity should be considered at many stages of the fibers’ toxicokinetic behavior.

Fibrous forms of the mineral talc are found in almost all talc deposits. The sample we report is a talc fiber concentrate from the Gouverneur Talc District of New York. The district is known for the occurrence of both talc and talc-amphibole fibers occurring in an asbestiform habit. The talc mines of the Gouverneur district contain mineral fiber that varies in abundance, composition, and mineralogy, including variations in the abundance and composition of the amphibole component of the fiber. The higher the amphibole component in a talc-amphibole fiber, the more rod-like are the fibers, the higher their Young’s modulus, and the higher the likelihood is for biologically rigid fiber. However, as we demonstrated, fibrous talc samples have a combination of rigidity, dimensions, and biosolubility that make their mesothelioma potency negligible. It corresponds to numerous studies confirming absence of mesothelioma risk elevation in cohorts of cosmetic talc miners (Ierardi and Marsh, 2020).

It is a limitation of our study that the calculation of the rigidity index rests on many assumptions, introducing uncertainties in its usefulness for characterizing mesotheliomagenic populations. First, we do not know the exact Young’s modulus for the MWCNT used in the animal studies. Furthermore, Young’s modulus may vary with size of the particle measured and the method of measurement. All the values of Young’s modulus we used for minerals were determined from hand-sized samples, but for MWCNT, the values were determined by electron microscopy. To understand the effect of the possible range in Youngs modulus, we determined RI assuming the Young’s modulus of all MWCNT was 160 GPa. This resulted in an estimated RI for biological rigidity of 0.02 instead of 0.05 µm^2^ x GPa x 10^4^. To reflect this uncertainty, RI for biological rigidity could be expressed as between 0.02 and 0.1 µm^2^ x GPa x 10^4^. Second, we do not know the thickness of any of the particles in the database from direct measurement but assume all carbon nanotubes are cylindrical and asbestos thickness/width can be modeled reliably from a limited number of studies. Third, we assume averages are representative and can explain outcomes although populations identified by average values may vary in range, especially in the range of length. Characterization that contained this additional information might be informative in explaining observed variances in expected outcome. Despite these uncertainties, the data make a compelling case that rigidity is a critical variable in mineral fiber carcinogenicity.

## Data Availability

The original contributions presented in the study are included in the article/supplementary material, further inquiries can be directed to the corresponding author.

## Footnotes

1 “mineral-like” refers to particles that are composed of a fabricated material that has the atomic structure and major element chemical composition that is the same as a naturally occurring mineral.

2 While we focus on the physical properties of carbon fibers and mineral particles, we recognize that once at the target tissue, biochemical and biological processes at the mineral-cell interface ultimately result in the initiation of cancer, and these processes will likely vary among fiber types in important ways independent of dimension, especially with respect to processes that occur at the particle’s surface.
